# Dynamics of coral‐associated microbiomes during a thermal bleaching event

**DOI:** 10.1002/mbo3.604

**Published:** 2018-03-23

**Authors:** Wirulda Pootakham, Wuttichai Mhuantong, Lalita Putchim, Thippawan Yoocha, Chutima Sonthirod, Wasitthee Kongkachana, Duangjai Sangsrakru, Chaiwat Naktang, Nukoon Jomchai, Nalinee Thongtham, Sithichoke Tangphatsornruang

**Affiliations:** ^1^ National Center for Genetic Engineering and Biotechnology (BIOTEC) National Science and Technology Development Agency Pathum Thani Thailand; ^2^ Phuket Marine Biological Center Phuket Thailand

**Keywords:** 16S rRNA sequencing, coral microbiome, coral‐associated bacteria, heat stress, PacBio sequencing, *Porites lutea*, thermal bleaching

## Abstract

Coral‐associated microorganisms play an important role in their host fitness and survival. A number of studies have demonstrated connections between thermal tolerance in corals and the type/relative abundance of *Symbiodinium* they harbor. More recently, the shifts in coral‐associated bacterial profiles were also shown to be linked to the patterns of coral heat tolerance. Here, we investigated the dynamics of *Porites lutea*‐associated bacterial and algal communities throughout a natural bleaching event, using full‐length 16S rRNA and internal transcribed spacer sequences (ITS) obtained from PacBio circular consensus sequencing. We provided evidence of significant changes in the structure and diversity of coral‐associated microbiomes during thermal stress. The balance of the symbiosis shifted from a predominant association between corals and Gammaproteobacteria to a predominance of Alphaproteobacteria and to a lesser extent Betaproteobacteria following the bleaching event. On the contrary, the composition and diversity of *Symbiodinium* communities remained unaltered throughout the bleaching event. It appears that the switching and/or shuffling of *Symbiodinium* types may not be the primary mechanism used by *P. lutea* to cope with increasing seawater temperature. The shifts in the structure and diversity of associated bacterial communities may contribute more to the survival of the coral holobiont under heat stress.

## 1 INTRODUCTION

Coral reefs are among the most biologically diverse and economically important ecosystems on the planet, providing shelter to over 25% of all marine species (Moberg & Folke, 1999). Reef‐building corals are associated with a dynamic and highly diverse consortium of microorganisms including algae, bacteria, archaea, fungi, and viruses (Marhaver, Edwards, & Rohwer, 2008; Rosenberg, Koren, Reshef, Efrony, & Zilber‐Rosenberg, 2007). The coral animal and its associated microbes together comprise a holobiont (Knowlton & Rohwer, 2003; Rohwer, Seguritan, Azam, & Knowlton, 2002). Coral‐associated microorganisms play an important role in their host fitness and survival. The best‐known coral symbionts, phototrophic dinoflagellates of the genus *Symbiodinium*, contribute approximately 95% of the coral energy requirements (Muscatine, Falkowski, Porter, & Dubinsky, 1984). Besides conferring the ability to fix carbon to the coral holobiont, *Symbiodinium* have been shown to influence the resilience of their hosts to thermal stress (Boulotte et al., 2016; Hume et al., 2016; Keshavmurthy et al., 2014). Attempts to understand the differences in the coral host responses to elevated water temperature have largely focused on genetic variation of *Symbiodinium*. Given the capacity of reef‐building corals to host different types of algal symbionts, two potential adaptive mechanisms to ocean warming have been proposed. The first mechanism, symbiont “shuffling,” is an approach by which corals resist or recover from heat stress through an adjustment in relative abundance of *Symbiodinium* types already *in‐hospite* (Baker, Starger, McClanahan, & Glynn, 2004; Jones, Berkelmans, van Oppen, Mieog, & Sinclair, 2008; Rowan, 2004). The second mechanism, symbiont “switching,” takes place when the coral hosts acquire new types of *Symbiodinium* through uptake from the environment such as water column and sediments (Boulotte et al., 2016; Fautin & Buddemeier, 2004). In addition to the proposed switching/shuffling mechanisms, Howells et al. (2012) demonstrated that adaptive differences in heat sensitivity/tolerance existed not only among different types of *Symbiodinium*, but also among locally adapted populations belonging to a single *Symbiodinium* type (Howells et al., 2012). Additionally, a study by Bellantuono, Granados‐Cifuentes, Miller, Hoegh‐Guldberg, and Rodriguez‐Lanetty (2012), Bellantuono, Hoegh‐Guldberg, and Rodriguez‐Lanetty (2012) revealed that short‐term preconditioning of *Acropora millepora* to thermal stress allowed the corals to acclimatize to subsequent heat stress without any change in the make‐up on their symbiont types (Bellantuono, Hoegh‐Guldberg et al., 2012).

Corals have been shown to associate with a highly diverse group of bacteria that contribute important functions to the coral holobiont (Ainsworth et al., 2015; Bourne, Morrow, & Webster, 2016; Kim, 2006; Pootakham et al., 2017). Members of coral‐associated bacterial communities appear to be involved in the provisioning and cycling of carbon, nitrogen and sulfur in coral reefs (Lema, Willis, & Bourne, 2012; Lesser et al., 2007; Rädecker et al. 2015; Raina, Tapiolas, Willis, & Bourne, 2009; Wegley, Edwards, Rodriguez‐Brito, Liu, & Rohwer, 2007). These prokaryotes may also function in protecting their hosts against pathogenic microbes by preventing their colonization through physical occupation of otherwise available niches (Ritchie & Smith, 2004) or through the production of antibacterial compounds (Krediet, Ritchie, Paul, & Teplitski, 2013). A study by Gilbert, Hill, Doblin, and Ralph (2012) revealed that an intact bacterial consortium provided the coral holobiont resilience against thermal stress. The authors applied antibiotics to artificially manipulate the resident bacterial community and demonstrated that undisturbed bacterial consortium ameliorated the thermal stress response and promoted the recovery of their hosts from bleaching events (Gilbert et al., 2012). More recently, microbiome dynamics has been shown to be linked to patterns of coral heat tolerance (Ziegler, Seneca, Yum, Palumbi, & Voolstra, 2017). The authors demonstrated that the composition of bacterial community associated with *Acropora hyacinthus* was different across thermally variable habitats, and the community adapted to the new environment when corals were reciprocally transplated. Changes in coral‐associated bacterial communities during a bleaching event have also been documented in *A. millepora* from the Great Barrier Reef (Bourne, Iida, Uthicke, & Smith‐Keune, 2008) and *Acropora muricata* in Nan‐wan, Taiwan (Lee, Davy, Tang, & Kench, 2016). In one of the studies, the denaturing gradient gel electrophoresis (DGGE) patterns revealed a correlation between increasing seawater temperature and the appearance of *Vibrio*‐affiliated sequences (Bourne et al., 2008).

The scleractinian coral *Porites lutea* is one of the dominant reef builders widely distributed in the Indo‐West Pacific (Yeemin et al., 2009). Over the past two decades, a number of bleaching events have been reported in the Gulf of Thailand and Andaman Sea (Phongsuwan et al., 2013). The resilience of coral holobionts to environmental stressors, including elevated seawater temperature, appears to be strongly influenced by members of their associated microbial communities. Even though the important contribution of coral‐associated bacteria to the overall fitness and long‐term survival of coral hosts have been demonstrated in several species (Ainsworth et al., 2015; Bourne et al., 2008; Gilbert et al., 2012; Tout, Siboni et al., 2015; Vega Thurber et al., 2009; Ziegler et al., 2017), there has not been a study that examines the shifts in bacterial and algal communities associated with *P. lutea* during a thermal stress.

Here, we investigate the dynamics of coral‐associated microbiomes during a recent thermal bleaching event in the Andaman Sea. We employed a long‐read PacBio SMRT sequencing technology to obtain full‐length 16S rRNA and internal transcribed spacer (ITS) sequences and thoroughly examined the structure and dynamics of microbial communities associated with *P. lutea* prior to, during and following a bleaching event in 2016. While there were no significant changes in the structure and diversity of coral‐associated *Symbiodinium*, we observed that the bacterial communities became more diverse as their coral hosts experienced elevated water temperature. The balance of the symbiosis shifted from a predominant association between *P. lutea* and Gammaproteobacteria to a predominance of Alphaproteobacteria following the bleaching event. The comparison of bacterial community compositions and potential shifts in microbiomes between pre‐bleaching, bleaching, and post‐bleaching corals may be useful in bridging the knowledge gap on how these associated bacteria contribute to the resilience of their coral host to thermal bleaching.

## 2 EXPERIMENTAL PROCEDURES

### 2.1 Field sample collection and temperature monitoring

**Figure 1:**
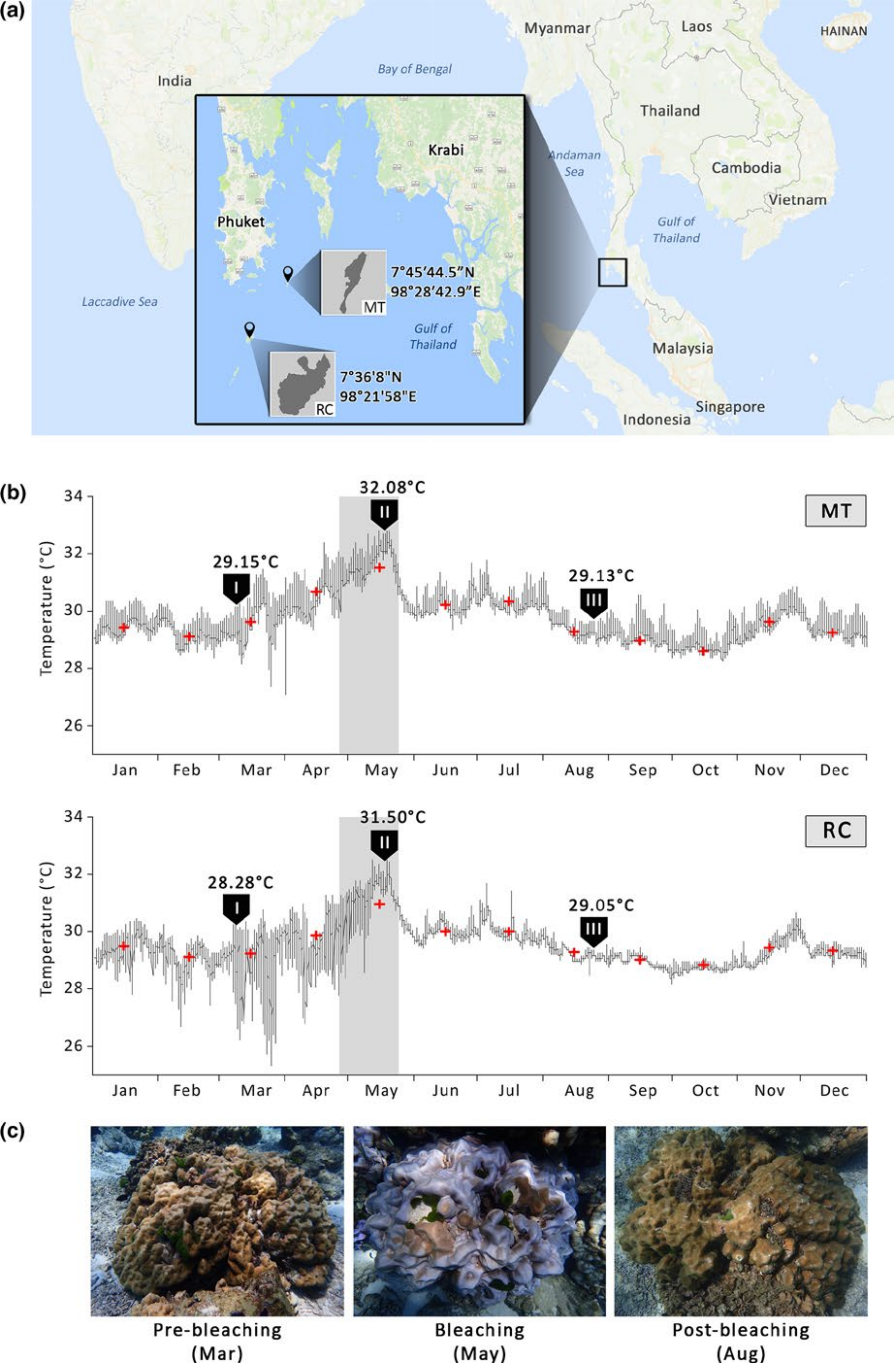
Coral sampling sites and temperature records. (a) A map displaying the sampling locations (along with GPS coordinates) in the Andaman Sea: Maiton (MT) and Racha (RC) islands. The main and inset maps were drawn based on the information from the Google Maps (Map data ©2017 Google). (b) Daily seawater temperatures at the sampling sites (MT and RC) from January to December 2016. Shading represents the bleaching event in the Andaman Sea in May. Arrows (I), (II) and (III) indicate the time points when samples were collected prior to (Mar), during (May) and following (Aug) the bleaching event, respectively. Average temperatures on the collection dates are shown above each sampling time point. Red crosses indicate monthly average temperatures. (c) Representative pictures of colonies collected in March (visually healthy), May (severely bleached), and August (visually healthy)

*P. lutea* samples were collected at the depth of 7 m from two locations in the Andaman Sea: Maiton (MT; 7°45′44.5″N 98°28′42.9″E) and Racha islands (RC; 7°36′8″N 98°21′58″E; Figure 1a). Three and four colonies were tagged at MT and RC, respectively, and coral samples were collected during the following stages: pre‐bleaching (March), bleaching (May), and post‐bleaching (August) in 2016. Coral colonies were visually healthy in March and August, but displayed signs of color loss in >80% of the colony surface area during the bleaching period in May (Figure 1c). One nubbin from each coral colony was collected underwater using scalpel blades, placed in sterile 2‐ml screw‐capped tubes without any air space and transported back to shore within 1–2 hrs in a 4°C container under low light. Seawater was immediately removed from the tubes upon returning to shore, and coral samples were submerged in liquid nitrogen and stored at −80°C prior to DNA extraction. Seawater temperatures at the depth of 7 m were recorded at a 20 min logging interval using Tidbit v2 Water Temperature Data Logger (Onset Computer Corporation, Bourne, MA) during the period between 1 January 2016 and 31 December 2016.

### 2.2 DNA extraction, 16S rRNA gene and ITS amplification, and PacBio sequencing

Tissue samples were homogenized in liquid nitrogen with sterile mortars and pestles, and genomic DNA was extracted, using the High Pure Template PCR Preparation Kit (Roche Life Science, Indianapolis, IN). DNA was eluted in 50 μl of 10 mmol/L Tris‐HCl pH 8.5 and quantified using the NanoDrop ND‐1000 Spectrophotometer (Thermo Fisher Scientific, Waltham, MA). DNA samples were diluted to 50 ng/μl for PCR amplification. Full‐length 16S rRNA sequences were amplified from 50 ng of genomic DNA template using two bacterial‐specific primers 27F (5′‐AGAGTTTGATCMTGGCTCAG) and 1492R (5′‐TACGGYTACCTTGTTACGACTT). *Symbiodinium* ITS was amplified using symITS1‐FP (5′ CTCAGCTTCTGGACGTTGYGTTGG 3′) and ITS2REV2 (5′ CCTCCGCTTACTTATATGCTT 3′) (van Oppen, Palstra, Piquet, & Miller, 2001). In order to multiplex amplicons from several samples into a single SMRTbell library, we employed a previously described two‐step PCR approach to add unique identifier (barcode) sequences to 16S rRNA/ITS amplicons from individual samples (Pootakham et al., 2017). First rounds of PCR were carried out using M13‐tagged forward (27F or symITS1‐FP) and reverse (1492R or ITS2REV2) primers in a final volume of 20 μl consisting of 0.4 U of Phusion High‐Fidelity DNA Polymerase (Thermo Fisher Scientific), 1x Phusion HF Buffer, 0.2 mmol/L dNTPs, 1.5 mmol/L MgCl_2_, 0.1 μmol/L of each primer and distilled water to make the remainder of the 20 μl volume. Conditions used for amplification in the thermocycler were as follows: preincubation at 98°C for 2 min, followed by 10 cycles of denaturation at 98°C for 30 s, annealing at 66°C for 15 s, elongation at 72°C for 45 s and 10 cycles of denaturation at 98°C for 30 s, annealing at 68°C for 15 s, elongation at 72°C for 45 s and a final extension step at 72°C for 5 min. Forward and reverse barcode sequences were added to 16S rRNA/ITS amplicons during the second round of PCR using M13F and M13R primers tailed with 16‐base PacBio barcodes at their 5′ ends. PacBio barcode sequences are available from https://github.com/PacificBiosciences/Bioinformatics-Training/blob/master/barcoding/pacbio_384_barcodes.fasta. PCR products from the first rounds of amplification were diluted 100‐fold in water, and 1 μl of the diluted templates were used in the secondary amplification reactions, which were carried out in a final volume of 20 μl consisting of 0.4 U of Phusion High‐Fidelity DNA Polymerase (Thermo Fisher Scientific), 1x Phusion HF Buffer, 0.2 mmol/L dNTPs, 1.5 mmol/L MgCl_2_, 0.1 μmol/L of each primer and distilled water to make the remainder of the 20 μl volume. Conditions used for amplification in the thermocycler were as follows: preincubation at 98°C for 2 min, followed by 3 cycles of denaturation at 98°C for 30 s, annealing at 63°C for 15 s, elongation at 72°C for 45 s and 5 cycles of denaturation at 98°C for 30 s, annealing at 66°C for 15 s, elongation at 72°C for 45 s and a final extension step at 72°C for 5 min.

Final PCR products were purified, using Agentcourt AMPure XP magnetic beads (Beckman Coulter, Indianapolis, IN) and quantified using the Qubit 2.0 Fluorometer and the Qubit dsDNA BR Assay Kit (Thermo Fisher Scientific). Purified 16S/ITS amplicons were pooled in equimolar concentrations, and 500 ng of DNA was used for library preparation. Since we were unable to extract high‐quality DNA from two samples (MT Mar [3] and RC Mar [4]) to amplify 16S rRNA and ITS fragments, the numbers of independent data points for each sample are as follows: MT Mar *n *=* *2; MT May, MT Aug and RC Mar *n *=* *3; RC May and RC Aug *n *=* *4. A total of two SMRTbell libraries were constructed (each containing amplicons from 9 to 10 samples) and sequenced on a PacBio RSII system, using the P6‐C4 polymerase and chemistry with a 360‐min movie time.

### 2.3 16S rRNA and ITS sequence data analysis

RS‐ReadsOfInsert protocol (SMRT Analysis software version 2.3) was used to demultiplex and process PacBio raw reads to obtain consensus sequences with a minimum of five full passes. ITS consensus reads shorter than 650 nt were removed (in order to exclude sequences derived from partial amplification) prior to downstream analyses. Filtered sequences were aligned to the ITS database (Tong et al., 2017) using BLASTN with an E‐value cutoff of 10^−5^ and the clade/subclade identification was assigned based on 97% sequence similarity. An OTU table was subsequently generated from the clade/subclade alignment data, and alpha diversity analyses were performed using the “vegan” package in R (Oksanen, Blanchet, Kindt, Legendre, & Minchin, 2013). The analysis of similarities (ANOSIM) was used to compare community assemblages based on the Bray‐Curtis distance matrix as implemented in the vegan function Adonis (permutation = 1000) (Oksanen et al., 2013).

For 16S rRNA CCS reads, we filtered out consensus sequences that were shorter than 1000 nt (in order to exclude sequences derived from partial amplification), and chimeric detection was performed on both strands, using the abundance‐based algorithm implemented in UCHIME (Edgar, 2010) and a reference dataset from RDP (Cole et al., 2014). Filtered sequences were analyzed using QIIME software version 1.9.1 (Caporaso et al., 2010). Once the close‐reference OTU picking was finished, the remaining sequences were clustered into OTUs based on an open‐reference OTU picking method at 97% identity, using UCLUST (Edgar, 2010). A representative sequence for each OTU was selected, and taxonomy was assigned, using the RDP Classifier (Wang, Garrity, Tiedje, & Cole, 2007) retrained toward the Greengenes database (version 13.8) (DeSantis et al., 2006). Alpha and beta diversity analyses were performed using the QIIME pipeline. The OTU table was rarefied to an even depth of 8022 sequences per sample (corresponding to the number of sequences present in the smallest sample) in order to avoid biases from unequal sampling depth. ANOVA and Tukey's honestly significant difference (HSD) post hoc tests were used to test for differences in relative abundance of associated bacteria between samples collected in March, May, and August. A nonparametric permutational multivariate analysis of variance (PERMANOVA) was used to compare community assemblages based on the unweighted Unifrac distance matrix as implemented in the vegan function Adonis (permutation = 1000) (Oksanen et al., 2013). The PacBio 16S rRNA and ITS sequence data were deposited in the GenBank NCBI Sequence Read Archive (SRA) database under the accession number PRJNA392355.

### 2.4 Performance comparison between full‐length and partial 16S rRNA sequences in species identification

To compare the ability of full‐length 16S rRNA, V3‐V4 and V5‐V6 sequences in species identification, we followed the protocol from Pootakham et al. (2017). First, we extracted the V3‐V4 and V5‐V6 hypervariable regions from the full‐length sequences using the same flanking sequences reported in Pootakham et al. (2017). The full‐length 16S rRNA sequences along with their respective V3‐V4 and V5‐V6 regions were subsequently aligned to the non‐redundant RDP reference (bacterial type strain) sequences using BLAST with an E‐value cutoff of 10^−10^. Any query sequence that returned two or more best hits belonging to different species with identical E‐value, bit‐score and aligned region was considered ineffective in resolving taxonomic classification at the species level. For taxonomic classification at the genus level, any query sequence that had two or more best hits belonging to different genera was considered “unassigned.”

### 2.5 Identification of *P. lutea* core microbiome

Candidate members of the *P. lutea* core microbiome were identified at 75% sample coverage (present in at least 15 out of 19 coral microbiome samples) based on previous studies (Hadaidi et al., 2017; Lawler et al., 2016; van de Water et al., 2016). Sample coverage is defined as the minimal percentage of all samples in which an OTU must be present to be considered part of the core microbiome. Bacterial type strain sequences were downloaded from RDP and clustered at 100% identity using the CD‐HIT software (Fu, Niu, Zhu, Wu, & Li, 2012) to obtain a set of nonredundant reference sequences. To identify candidate members of the core microbiome, full‐length 16S rRNA sequences were aligned to the nonredundant RDP reference sequences, using BLAST with a sequence identity cutoff of >90%.

### 2.6 Functional profiling of bacterial communities

We employed a computational approach, PICRUSt (phylogenetic investigation of communities by reconstruction of unobserved states), to predict the functional composition of bacterial communities using 16S rRNA marker data and a database of reference genomes (Langille et al., 2013). We first applied the command “normalize_by_copy_number.py” to the OTU abundance table to account for differences in gene copy number. Functional predictions of KEGG Orthologous groups (KOs) were carried out using the command “predict_metagenomes.py,” and the KOs were summarized to KEGG pathways (level 1, 2, 3) with “categorize_by_function.py.” The LEfSe method (Segata et al., 2011) was used to identify significantly different metagenome functions of bacterial communities among samples collected before, during and following the bleaching event (LDA > 3.0).

## 3 RESULTS AND DISCUSSION

### 3.1 Assessment of *P. lutea*‐associated bacterial diversity during a thermal bleaching event

Full‐length 16S rRNA gene and ITS sequences were amplified from microbial communities associated with *P. lutea* collected from two locations in the Andaman Sea: Maiton (MT) and Racha (RC) islands (Figure 1a). Corals were collected from each sampling site during the following periods: March (pre‐bleaching), May (bleaching) and August (post‐bleaching; Figure 1b,c). During the period from 9 May to 22 May, daily average temperatures exceeded 31.5°C and peaked at 32.8°C and 32.5°C in MT and RC, respectively (Figure 1b).

A total of 1013,753 and 424,500 PacBio raw reads totaling 25.98 and 7.71 Gb were obtained from 16S rRNA and ITS sequences, respectively. Polymerase reads were assembled and demultiplexed into 295,955 16S rRNA and 141,487 ITS circular consensus sequencing (CCS) reads (Tables S1 and S2). Following the removal of chimeras and 16S rRNA CCS reads shorter than 1000 nt, a total of 238,497 reads were obtained for all samples. The number of processed full‐length 16S rRNA sequences ranged from 8022 to 26,438 per sample, with an average of 12,552 reads/sample (Table S1). After removing ITS sequences that were shorter than 650 nt, the number of processed reads ranged from 986 to 10,583 per sample, with an average of 5449 reads/sample (Table S2).

**Figure 2:**
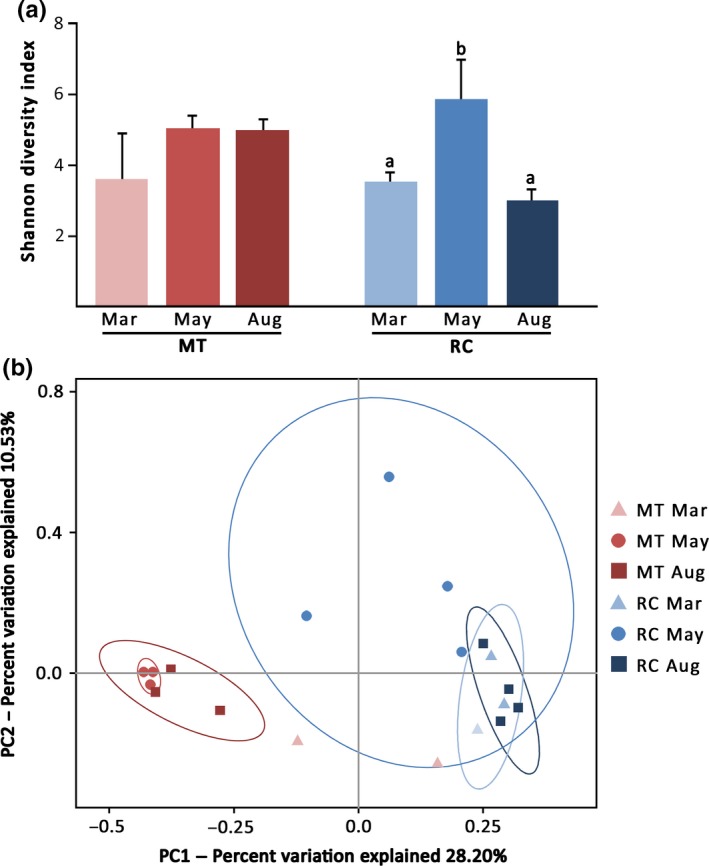
Alpha and beta diversity estimates of bacterial communities associated with *P. lutea* in MT and RC prior to (Mar), during (May), and following (Aug) the bleaching event. (a) A bar chart displaying Shannon diversity indices of coral‐associated microbiomes. Bars with different superscript letters (a,b) denote significant differences among RC samples (Tukey's HSD;* p *<* *.05). Numbers of independent data points for each sample are as follows: MT Mar *n *=* *2; MT May, MT Aug and RC Mar *n *=* *3; RC May and RC Aug *n *=* *4. Shannon diversity indices were not significantly different among MT samples. (b) Principal coordinate analysis (PCoA) was used to plot the beta diversity of bacterial communities using the unweighted Unifrac Matrix. Red symbols indicate samples collected from MT whereas blue symbols indicate samples collected from RC. The ellipses indicate 95% confidence intervals of each sample group (note that that confidence interval could not be drawn for MT‐Mar group since *n *<* *3)

To evaluate the diversity of bacterial community present within each sample and among samples, alpha and beta diversity indices were calculated. The highest number of OTUs was observed in RC May (4) sample (1,056 OTUs; Table S1). Rarefaction curves seemed to plateau off for most samples, suggesting that sufficient sampling has been performed to capture the total diversity of the microbiomes (Figure S1). In MT, pre‐bleaching corals (Mar) appeared to harbor bacterial communities with lower degree of diversity than those exposed to elevated seawater temperature (May and Aug; Figure 2a). A significant increase in the Shannon index between pre‐bleaching (Mar) and beaching (May) samples was also observed in RC; however, in contrast to MT, the diversity of bacterial community associated with post‐bleaching corals in RC returned to the level observed prior to the bleaching event. Principal coordinate analysis (PCoA) of unweighted UniFrac distances based on Ribosomal Database Project (RDP) results showed that MT and RC samples harbored different bacterial communities throughout the bleaching event (Figure 2b). The bacterial profiles of bleaching MT‐May samples clustered with the profiles of post‐bleaching MT‐Aug samples, and even though MT‐Mar sample size was small (*n *=* *2), the bacterial profiles of both MT‐May and MT‐Aug were significantly different from that of MT‐Mar. On the other hand, the associated bacterial profiles of RC‐Mar corals clustered with those of RC‐Aug corals, and these two profiles were distinct from microbiome profiles of bleaching RC colonies sampled in May (Figure 2b).

Similar to our observations in *P. lutea* corals, an increase in bacterial diversity during a bleaching period has previously been reported in *A. millepora* (Bourne et al., 2008) and *A. muricata* (Lee et al., 2016). Bourne et al. (2008) also noticed a return to pre‐bleaching diversity levels of coral‐associated bacterial communities as *A. millepora* colonies recovered from the thermal stress, similar to the phenomenon observed with RC corals in our study (Figure 2a). Interestingly, the bacterial diversity of post‐bleaching samples in MT remained relatively high, comparable to that of bleaching corals despite the fact that post‐bleaching samples from both locations were from coral colonies that appeared visually healthy (Figure 2a). The difference suggests that local environmental conditions have a profound impact on the structure of coral‐associated bacterial communities and how quickly these communities recover after the exposure of a coral holobiont to thermal stress.

### 3.2 Distinct shifts in *P. lutea*‐associated bacterial communities during a heat stress

**Figure 3:**
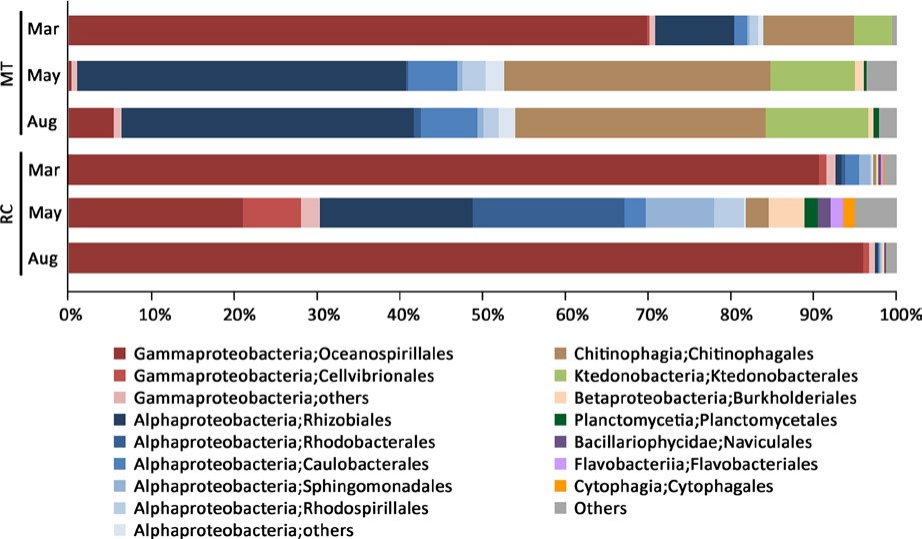
Composition of bacterial communities associated with *P. lutea* prior to (Mar), during (May) and following (Aug) a thermal bleaching event in MT and RC. Depicted is taxonomic classification of OTUs present in each sample group at the class/order levels based on Greengenes database, using QIIME software. Fourteen most abundant orders from nine classes are plotted, and the remaining taxa are grouped under “others”

A total of 229,982 out of 238,497 filtered reads from both MT and RC were assigned using the RDP classifier (Wang et al., 2007) with a confidence threshold of 80%. Sequences were classified into 18 phyla (13 known and 5 candidate phyla), 44 classes and 85 orders present across all samples (Dataset 1). Even though Proteobacteria represented the ubiquitous and predominant taxon in all MT and RC samples, the structure and diversity of *P. lutea*‐associated microbiomes changed dramatically when corals experienced the thermal bleaching in May. Compared to pre‐bleaching samples, Gammaproteobacteria order Oceanospillales became significantly less abundant in corals collected during the bleaching period in both locations (Tukey's HSD, *p *<* *.05; Figure 3). Concomitantly, Alphaproteobacteria orders Rhizobiales, Rhodobacteriales, Caulobacteriales, and Rhodospillales increased in their relative abundances during the warm seawater temperature period. Additionally, the proportions of Betaproteobacteria (Burkholderiales) and Planctomycetia (Planctomycetales) present in samples collected in May increased significantly compared to those collected in March (Tukey's HSD, *p *<* *.05). Intriguingly, the shifts in bacterial profiles associated with bleaching samples to those associated with post‐bleaching samples were completely different between the two locations. While a change in bacterial community structure between bleaching and post‐bleaching samples in MT was negligible (PERMANOVA, pseudo‐*F *=* *1.08, *p *=* *.395), significant shifts in bacterial communities were observed between bleaching and post‐bleaching corals retrieved in RC (PERMANOVA, pseudo‐*F *=* *1.57, *p *=* *.024; Figures 2b and 3). The microbiome profiles of post‐bleaching RC‐Aug samples shifted to become more similar to profiles of pre‐bleaching RC‐Mar samples (Figure 3).

**Figure 4:**
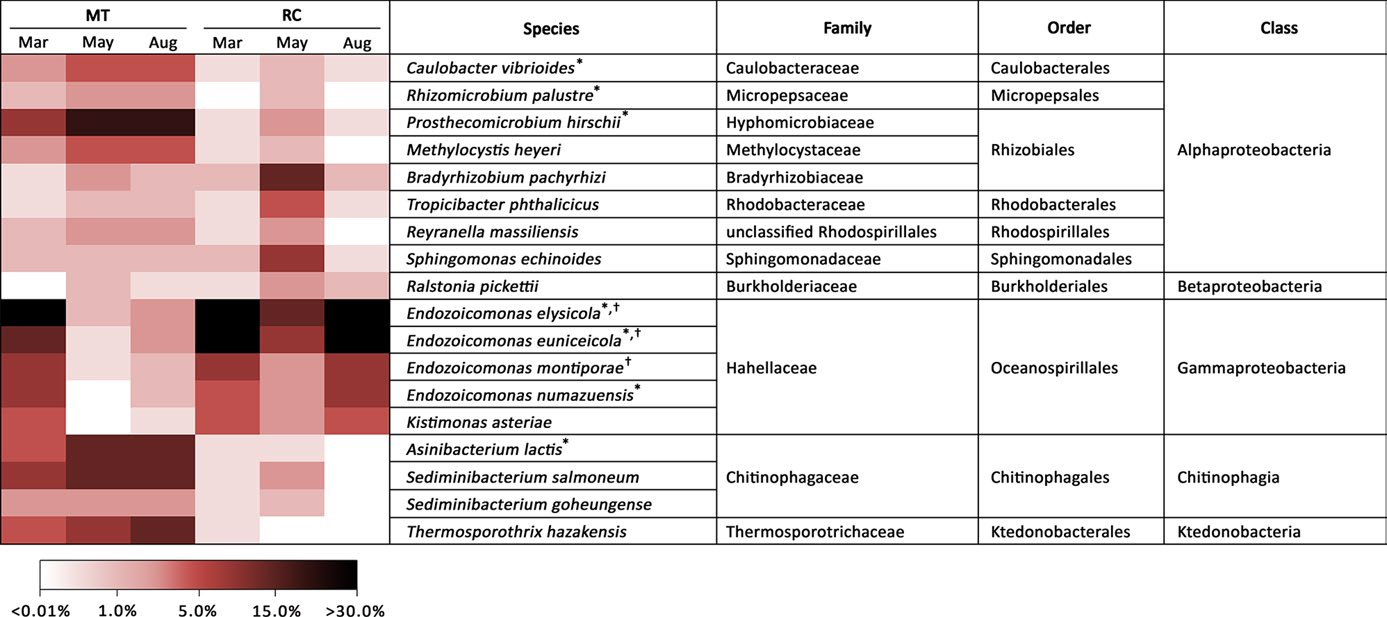
Distribution of 18 most prevalent bacterial species associated with *P. lutea* samples from MT and RC before (Mar), during (May) and after (Aug) a thermal bleaching event in the Andaman Sea. An asterisk (*) and a dagger (†) denote significant differences (Tukey's HSD;* p *<* *.05) among samples from MT and RC, respectively

Full‐length 16S rRNA sequence data allowed us to identify members of *P. lutea* microbiomes at the species resolution. The abundances of several *Endozoicomonas* species *(E. elysicola, E. euniceicola, E. montiporae,* and *E. numazuensis*) averaged across all 19 samples appeared to be significantly different among corals collected from different bleaching status (Tukey's HSD, *p *<* *.05; Figure 4). In addition, a few other Alphaproteobacteria (*Caulobacter vibrioides, Rhizomicrobium palustre* and *Prosthecomicrobium hirschii*) and a member of Chitinophagia (*Asinibacterium lactis*) were significantly different in their relative abundances among corals sampled before, during and after the thermal stress (Tukey's HSD, *p *<* *.05; Figure 4).

### 3.3 Stability of *P. lutea*‐associated *Symbiodinium* communities throughout a bleaching event

In contrast to the shifts in bacterial community observed when the corals were under heat stress, minimal changes were observed in *Symbiodinium* community structures (Figure S3). The profile of associated *Symbiodinium* remained essentially the same across samples from different locations and temperature conditions (ANOSIM on Bray‐Curtis distance matrix, *p *=* *.626). Consistent with previous investigations (Hume et al., 2015; Tanzil et al., 2016; Yamashita, Suzuki, Hayashibara, & Koike, 2011), only members of *Symbiodinium* clade C were detected in our *P. lutea* samples collected from both MT and RC islands. Most of the ITS sequence reads (75%) were predominantly affiliated with subclade C116, and the remaining sequences belonged to subclades C15f, C15g, C15h, C15.2, C15.6, and C15.7 (Figure S3). Even though ITS sequences are the most widely used genetic marker for assessing the diversity of *Symbiodinium* community, it is worthwhile to mention the caveat of this marker. The multicopy nature and known variability of ITS sequences within individual *Symbiodinium* cells render it nearly impossible to accurately identify the number of biological entities the sequence data represent (Stat et al., 2011). The presence of intragenomic variations in ITS can potentially confound estimates of microbial diversity in a community (Thornhill, Lajeunesse, & Santos, 2007). Nevertheless, the consequences of under‐ or overestimating the proportion of each *Symbiodinium* clade/subclade and the diversity indices in our study are unlikely to affect our conclusion that there was no significant change in the coral‐associated *Symbiondinium* community structure during a thermal bleaching event.

*P. lutea* is one of the coral species in which eggs or larvae acquires *Symbiodinium* from their female parents through a vertical transmission (Baird, Guest, & Willis, 2009). Vertically transmitted symbiont communities are often found in brooding corals with internal fertilization and are hypothesized to be of lower diversity and higher fidelity (Baird et al., 2009). A low degree of diversity observed in *P. lutea*‐associated *Symbiodinium* may be due to this direct transfer of symbionts from parents to offspring (Byler, Carmi‐Veal, Fine, & Goulet, 2013). This type of symbiont inheritance may reduce the likelihood of progeny obtaining new type/clade of *Symbiodinium* from the benthos.

Several studies have demonstrated shifts in *Symbiodinium* clades, by either switching or shuffling less heat tolerant for more heat tolerant types, enabling acclimation of coral holobiont to increasing seawater temperatures (Boulotte et al., 2016; Jones et al., 2008; Rowan, 2004). Boulotte et al. (2016) provided evidence supporting de novo acquisition of thermally resistant *Symbiodinium* clade D in *Pocillopora damicornis* and *Stylophora pistillata* following bleaching events. Another study demonstrated a dramatic shift in symbiont community from a predominant association between *A. millepora* and *Symbiodinium* type C2 prior to a bleaching event to a predominance of type D and type C1 after bleaching (Jones et al., 2008). Contrary to what was reported for other coral species (Boulotte et al., 2016; Jones et al., 2008; Rowan, 2004), the effect of increasing seawater temperature on the distribution of each subclade within the *P. lutea‐*associated algal community was negligible (PERMANOVA on Bray‐Curtis distance matrix, *F *=* *1, *p *=* *.412). The structure of *Symbiodinium* communities harbored by *P. lutea* from both MT and RC remained fairly stable throughout the bleaching event (Figure S3). Similar to earlier observations, we noticed that *P. lutea* collected in the Andaman Sea exclusively harbored *Symbiodinium* clade C (Hume et al., 2015; Tanzil et al., 2016; Yamashita et al., 2011). A plausible explanation for the absence of a symbiont partner shift in our study is that these hosts have already established an optimal symbiotic relationship with a specific consortium of *Symbiodinium* that allows the coral holobionts to survive and thrive in their local environments. After multiple occurrences of thermal bleaching in the past few decades (1991, 1995, 2003, and 2010), the specific association of *P. lutea* with *Symbiodinium* clade C may be an optimal one that allows both partners to survive an increase in water temperature (Chavanich, Viyakarn, Adams, Klammer, & Cook, 2012; Wall et al., 2015). Switching or shuffling *Symbiodinium* types may not be the primary mechanism used by this coral species to acclimate to increasing seawater temperature, and shifts in the structure and diversity of associated bacterial communities may contribute more to the survival of the coral holobiont under heat stress. Additionally, Bellantuono, Granados‐Cifuentes et al. (2012); Bellantuono, Hoegh‐Guldberg et al. (2012) has demonstrated that the *Symbiodinium* composition prior to heat treatments and after 8 days of thermal challenge was the same across both treatment and control samples (Bellantuono, Granados‐Cifuentes et al., 2012). Findings from Bellantuono, Granados‐Cifuentes et al. (2012); Bellantuono, Hoegh‐Guldberg et al. (2012) and our study suggest that physiological acclimatization of the host and symbionts is a significant part of the response to heat stress. Host physiology and ability to induce stress response through the upregulation of heat‐shock proteins has been suggested to play a role in heat stress tolerance in addition to harboring heat‐resistant symbiont (Fitt et al., 2009; Rodriguez‐Lanetty, Harii, & Hoegh‐Guldberg, 2009). To further investigate changes at the cellular level in both corals and their symbionts, future work on gene expression analysis in *P. lutea* during heat stress will be required.

### 3.4 Changes in *P. lutea‐*associated microbiomes during a heat stress

In contrast to an insignificant change in coral‐associated algal communities during heat stress, shifts in bacterial communities were evident when *P. lutea* were exposed to elevated seawater temperature. There was a marked decline in the apparent relative abundance of Gammaproteobacteria, particularly members of Oceanospirillales (Figure 3 and Figure S2). Concomitantly, the microbiome structure of bleaching corals shifted toward a predominance of Alphaproteobacteria (Rhizobiales, Rhodobacterales, Caulobacterales, Rhodospirillales) and to a lesser extent Betaproteobacteria (Burkholderiales). Similar to our observation, a shift from Oceanospirillales‐ to Rhodobacterales‐dominating community was observed over a 7‐day heat stress experiment (McDevitt‐Irwin, Baum, Garren, & Vega Thurber, 2017; Tout, Siboni et al., 2015). Tout, Jeffries et al. (2015); Tout, Siboni et al. (2015) also reported that *P. damicornis*‐associated bacterial community experiencing heat stress had significantly higher diversity. Furthermore, studies performed in *A. millepora* (Bourne et al., 2008) and *A. muricata* (Lee et al., 2016) demonstrated that bacterial community became more diverse as the coral hosts experienced thermal stress and that the community shifted from being dominated by Gammaproteobacteria to Alphaproteobacteria at higher temperatures. The thermal‐induced shift of bacterial population away from a stable microbiota appears to be a common response among scleractinian corals. Such changes in the bacterial communities are likely to affect the physiological function of these communities.

Lee et al. (2016) demonstrated that heat stress and bleaching have an impact on *A. muricata* mucus composition, causing a decrease in glucose, mannose and N‐acetylglucosamine (GluNAc) and an increase in arabinose, fucose, and N‐acetylgalactosamine (GalNAc). These changes in mucus sugar composition are likely to directly or indirectly influence the shifts in diversity and structure of bacterial populations during thermal stress. Members of Gammaproteobacteria (*E. montiporae, E. elysicola, Vibrio coralliilyticus*, and *Vibrio natriegens*) are capable of utilizing a wider range of sugars as their carbon source; however, only an increase in the relative abundance of *V. coralliilyticus* in thermally stressed *A. muricata* was observed, suggesting that factors besides mucus composition also influenced bacterial populations in the holobiont (Lee et al., 2016). A number of studies observed a prominent increase in relative abundance of *Vibrio*‐affiliated sequences during bleaching (Bourne et al., 2008; Lee et al., 2016; Tout, Siboni et al., 2015). *Vibrio* species are often acknowledged for their roles as opportunistic or pathogenic bacteria associated with coral diseases (Roder et al., 2014; Sweet & Bythell, 2012). Interestingly, significant shifts in the relative sequence abundance of Proteobacteria classes between healthy and white plaque disease affected tissues observed in *Diploria strigosa* and *Siderastrea siderea* were similar to the changes observed in coral tissues experiencing heat stress (Cardenas, Rodriguez, Pizarro, Cadavid, & Arevalo‐Ferro, 2012). Diseased tissues showed a consistent increase in Alphaproteobacteria (Rhodobacterales) associated with a decrease in Gammaproteobacteria (Oceanospiralles) compared to samples from healthy tissues. Despite the similarity in the bacterial profile shifts associated with disease and thermal bleaching, we did not observe an increase in relative abundance of *Vibrio* or other known coral pathogen species under elevated seawater temperature. As a matter of fact, *Vibrio*‐related sequences were barely detected in our samples across locations and time periods, suggesting that the shifts in coral‐associated microbiomes observed in this study were unlikely to be related to the effects of a pathogenic agent. The increase in relative abundance of Alphaproteobacteria may simply reflect an opportunistic colonization of this group of bacterial communities on coral tissues damaged from the heat stress. The reciprocal decrease in relative abundance of Gammaproteobacteria may favor the increase in Alphaproteobacteria by a loss of antagonism or other growth control mechanisms (e.g., antibiotic production) in nonstressed microbiomes.

Even though bacterial communities associated with *P. lutea* samples from MT and RC exhibited a similar shift from being Gammaproteobacteria‐dominated to Alphaproteobacteria‐dominated during heat stress, only the microbiomes associated with samples from RC appeared to recover and return to the structure and diversity observed prior to the bleaching event within 3 months (Figure 3 and Figure S2). In contrast, the profiles of MT‐Aug microbiomes remained similar to those of MT‐May samples, suggesting that the dynamics and the recovery of coral‐associated bacterial communities were affected by local environmental factors. Daily water temperatures between January to May appeared to fluctuate more strongly in RC compared to MT (Figure 1b). These high‐frequency step changes in temperature were likely due to large‐amplitude internal waves, which are strongest during the dry northeast monsoon season (January through March) (Wall et al., 2015). RC island is located on the continental shelf in the Andaman Sea and is exposed to large‐amplitude internal wave impact while MT island is sheltered from those waves (hence, less daily temperature fluctuation). A study by Wall et al. (2015) demonstrated that the cooling effect of large‐amplitude internal waves was beneficial to coral reefs during thermal stress as coral groups from the area exposed to large‐amplitude internal waves showed milder bleaching than those from the area sheltered from those waves. Even though MT and RC colonies chosen for this study exhibited similar bleaching profiles (signs of color loss in >80% of the colony surface area in May and completely recovered in August), RC coral‐associated microbiomes might benefit from the cooling effect of large‐amplitude internal waves in such a way that allowed them to return to the community structure observed prior to the bleaching event within a few months. It is also plausible that other environmental factors in RC (e.g., salinity and pH) influence the rate at which the structure and diversity of *P. lutea* microbiomes returned to the pre‐bleaching configuration. The composition of post‐bleaching microbiomes in MT may take longer to return to their original state. If we were to collect another set of samples toward the end of 2016, we might observe the microbial community structure that was similar to that of the pre‐bleaching samples. These results highlight the importance of local environmental factors on the dynamics of coral‐associated bacterial communities in response to thermal stress.

### 3.5 The *P. lutea* core microbiome

Despite the differences in microbial communities associated with *P. lutea* from MT and RC throughout the bleaching event, we examined the conserved bacterial species that were consistently present in at least 75% of the samples regardless of the relative abundance of each species. Following this criterion, we identified 17 species belonging to a diverse group of bacteria from 9 families, 6 orders and 4 classes as members of the core microbiome (Table 1). Gammaproteobacteria and Alphaproteobacteria constituted the greatest proportions of the core microbiomes, representing 63.51% and 27.33%, respectively (Table 1). Full‐length 16S sequences have an advantage of covering all hypervariable regions of the 16S rRNA genes and enable high‐resolution taxonomic classification of the core microbiome members at the species level. The majority of the conserved bacterial community belonged to the genus *Endozoicomonas*, with five species identified as members of the core microbiome (Table 1). Three highly conserved core members consistently present at 100% sample coverage across spatial and temporal variations were members of Hahellaceae (*E. elysicola, E. montiporae*) and Bradyrhizobiaceae (*Bradyrhizobium pachyrhizi*) families. Eleven out of seventeen species have also been identified as *P. lutea* core microbiome members in a previous study, and it is noteworthy to mention that *E. elysicola* have consistently been detected in all *P. lutea* samples collected from seven sampling sites in the Gulf of Thailand and Andaman Sea (Pootakham et al., 2017). Compared to other Oceanospirillales symbionts, *Endozoicomonas* genomes appear to be enriched for genes involved in transport activities, especially carbon sugar transport and the secretion of proteins (Neave, Michell, Apprill, & Voolstra, 2017). This enrichment in transport and secretion may allow efficient transfer of organic molecules between the symbionts and corals, suggesting that *Endozoicomonas* species play important roles in the upcycling or carbohydrates and the provision of proteins to the host (Neave et al., 2017). Furthermore, the ability of *E. montiporae* and *E. elysicola* to utilize a wide variety of sugars found in coral mucus (Lee et al., 2016) probably allows these two *Endozoicomonas* species to remain associated with *P. lutea* throughout the bleaching event, regardless of the change in coral mucus composition resulting from elevated seawater temperature.

**Table 1:** A list of bacteria present in *P. lutea* core microbiome (species that were found in at least 75% of all samples), their relative abundances (average abundance across all 19 samples) and their average ubiquity (defined as a percentage of *P. lutea* samples in which the species was detected)

Class	Order	Family	Genus/species	In this study		In previous study	
				Average abundance	Average ubiquity	Average abundance	Average ubiquity
Alphaproteobacteria	Caulobacterales	Caulobacteraceae	***Caulobacter henricii***	1.44%	79%	0.192%	89%
			***Caulobacter vibrioides***	5.94%	84%	2.657%	100%
			*Phenylobacterium kunshanense*	1.13%	74%	ND	ND
	Rhizobiales	Bradyrhizobiaceae	Bradyrhizobium pachyrhizi*	4.07%	100%	ND	ND
			*Oligotropha carboxidovorans*	0.02%	79%	ND	ND
		Hyphomicrobiaceae	***Prosthecomicrobium hirschii***	0.15%	89%	1.652%	100%
		Methylocystaceae	***Methylocystis heyeri***	9.05%	74%	0.115%	83%
	Rhodospirillales	Rhodospirillaceae	***Reyranella massiliensis***	5.53%	74%	3.881%	100%
Betaproteobacteria	Burkholderiales	Burkholderiaceae	*Ralstonia pickettii*	6.06%	89%	ND	ND
Chitinophagia	Chitinophagales	Chitinophagaceae	***Sediminibacterium salmoneum***	3.12%	84%	4.336%	100%
Gammaproteobacteria	Oceanospirillales	Hahellaceae	***Endozoicomonas elysicola***	0.33%	100%	21.340%	100%
			***Endozoicomonas euniceicola***	35.45%	95%	17.399%	100%
			Endozoicomonas gorgoniicola*	0.24%	74%	ND	ND
			***Endozoicomonas montiporae***	1.55%	100%	2.125%	94%
			***Endozoicomonas numazuensis***	24.60%	95%	3.637%	100%
			***Kistimonas asteriae***	0.61%	74%	2.292%	94%
		Oceanospirillaceae	*Neptunomonas antarctica*	0.73%	74%	ND	ND

Asterisks denote species that were not identified as parts of the core microbiome themselves in the previous study (Pootakham et al., 2017) but their closely related species in the same genus were. ND indicates that the species was not included as members of the core microbiome in the previous study. Species in bold fonts were identified as part of the *P. lutea* core microbiome in both studies.

The majority of species identified as members of the *P. lutea* core microbiome were present in low abundance (Table 1). Of 17 species found within at least 75% of all samples, 11 had a relative abundance lower than 5% within the whole community, including the three highly conserved core members (present at 100% sample coverage). This is consistent with previous findings that bacteria that form stable and species‐specific associations may be present at low relative abundance in the coral microbiome (Ainsworth et al., 2015; Hernandez‐Agreda, Gates, & Ainsworth, 2017; Littman, Willis, Pfeffer, & Bourne, 2009; Pootakham et al., 2017). In addition to the low abundant members of the core microbiome, we observed the presence of two highly abundant members, *E. euniceicola* and *E. numazuensis*, that represented 35.45% and 24.60% of the total microbiome, respectively. Several recent studies have shown that in gorgonian corals, members of core microbiome may be present in high abundance and constitute more than 50% of the total bacterial community (van de Water, Melkonian et al., 2017; van de Water, Voolstra et al., 2017).

### 3.6 Full‐length 16S rRNA sequence data allow taxonomical classification at the species level

**Figure 5:**
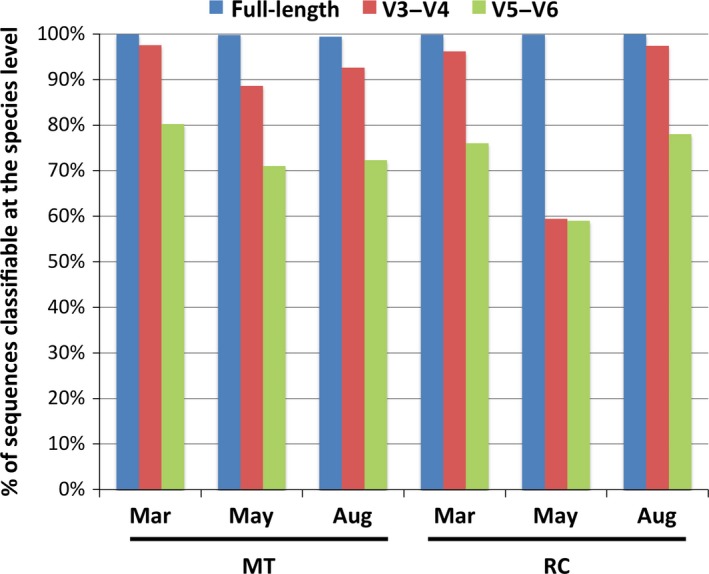
A bar chart illustrating the percentages of 16S rRNA sequence reads that are classifiable at the species level, using full‐length (blue) or partial amplicons (V3‐V4, red; V5‐V6, green)

In this study, we utilized the long‐read PacBio sequencing technology to capture full‐length 16S rRNA sequences to investigate the structure and diversity of coral‐associated bacterial communities during a bleaching event. To demonstrate a key advantage of using full‐length sequences in 16S rRNA gene‐based community surveys, we compared the resolution of microbial community analyses performed using short hypervariable regions and full‐length 16S rRNA sequences. We extracted the commonly used V3‐V4 and V5‐V6 regions from the full‐length reads and aligned both the in silico amplicons (420‐bp V3‐V4 and 255‐bp V5‐V6 fragments) and their respective full‐length sequences against non‐redundant reference sequences in the RDP (Cole et al., 2014). We observed marked differences in the proportions of sequences that could be classified at the species level using V3‐V4, V5‐V6 regions or full‐length 16S amplicons (Figure 5). Over 99.4% of the full‐length 16S rRNA sequences were taxonomically classified at the species level while 59%–98% and 59%–80% of the V3‐V4 and V5‐V6 sequences, respectively, could be assigned to specific species. These results showed that partial 16S rRNA sequence information from the hypervariable V3‐V4 or V5‐V6 regions is often insufficient for high‐resolution taxonomic assignments at the species level.

In addition, we compared taxonomic profiles (at the genus level) obtained from V3‐V4, V5‐V6 or full‐length 16S rRNA sequences (Table S3). Taxonomic classification based on short hypervariable V3‐V4 and V5‐V6 regions yielded different microbiome profiles from the classification performed, using full‐length 16S sequences. The number of unassigned sequences was also notably higher when partial 16S fragments were used instead of the full‐length sequences, indicating that these commonly used hypervariable regions were identical or nearly identical among several bacterial 16S rRNA sequences in the reference database. Uncertain classification or misclassification of V3‐V4 or V5‐V6 hypervariable regions can affect the accuracy of taxonomic profiles as well as bacterial community's estimates of species richness (Youssef et al., 2009). The estimates of OTUs and species richness can be under‐ or overestimated depending on the hypervariable fragments used, and the bias in species richness estimates observed can be explained by the proportions of hypervariable, variable, and conserved nucleotide positions present in the regions used to perform the analyses (Youssef et al., 2009). The utilization of full‐length 16S rRNA sequence information will help us avoid the issues of misclassification or classification discrepancy and the inflation of diversity indices, resulting from the use of short hypervariable fragments.

### 3.7 Microbial functional profiles change during thermal stress

To explore the relationship between functional genes of coral‐associated microbial communities and seawater temperatures, we employed a computational approach PICRUSt (Langille et al., 2013) to assess putative functional changes underlying the distinct microbial communities associated with corals during a heat stress (Table 2). The taxonomy‐based functional profiles of microbiomes associated with healthy corals showed an enrichment of several protein functions related to fundamental cellular processes such as carbohydrate metabolism, nucleotide synthesis, and fatty acid metabolism. Interestingly, the functional profiling of pre‐bleaching microbiomes also revealed multiple protein functions related to chemotaxis, including chemotaxis proteins CheR and CheW and motility proteins MotA and MotB (Table 2). Chemotactic behaviors among natural populations of coral‐associated prokaryotes have been demonstrated in situ, with high levels of activities toward a range of coral holobiont exudates such as amino acids and the organic sulfur compound dimethylsulfoniopropionate (DMSP) (Christian, Holger, & Markus, 2005; Raina et al., 2013; Tout, Jeffries et al., 2015). Tout, Jeffries et al., 2015; Tout, Siboni et al., 2015 observed a stronger chemotactic response in coral‐associated bacteria obtained from coral surface of *P. damicornis* than in noncoral associated bacteria collected from the open water, suggesting that chemotaxis may be involved in the establishment of specific coral‐bacterial relationships and may play a role in shaping the composition of coral reef microbial communities. Because coral surface microenvironments are characterized by strong gradients of chemical cues and organic molecules (Mass, Genin, Shavit, Grinstein, & Tchernov, 2010), motility and chemotaxis are likely to be important phenotypes favoring the colonization of corals by bacteria. The ability to use chemotaxis to locate nutrient‐rich surfaces likely provides advantages for reef‐associated microbes by allowing them access to otherwise limiting resources. Some of the earlier work on marine bacterial chemotaxis demonstrated that motility and chemotaxis are important features for the coral pathogens *V. shiloi* and *V. coralliilyticus* to locate and invade their host tissues (Banin, Israely, Fine, Loya, & Rosenberg, 2001; Meron et al., 2009; Rosenberg et al., 2007). More recently, Garren et al. (2014) demonstrated that *V. coralliilyticus* exhibited strong chemotactic responses toward DMSP released by its host *P. damicornis* during heat stress (Garren et al., 2014). However, since we did not detect pathogen‐related sequences in any of our microbiome samples, the enrichment of functional profiles related to chemotaxis observed here is likely associated with successful establishment and maintenance of specific coral‐bacteria associations, which ultimately influence the health and stability of the coral holobionts under optimal conditions.

**Table 2:** Enrichment of functional proteins in *P. lutea*‐associated microbial communities prior to (Mar) and during a heat stress (May)

KEGG orthology (KO)	Month	LDA score	*P‐*value	Functional annotation
K03406	Mar	3.899	0.023	Methyl‐accepting chemotaxis protein
K02013	Mar	3.314	0.035	Iron complex transport system ATP‐binding protein [EC:3.6.3.34]
K03408	Mar	3.226	0.035	Purine‐binding chemotaxis protein CheW
K06177	Mar	3.212	0.014	Ribosomal large subunit pseudouridine synthase A [EC:5.4.99.12]
K01825	Mar	3.203	0.021	3‐hydroxyacyl‐CoA dehydrogenase [EC:1.1.1.35]
K01187	Mar	3.128	0.039	Alpha‐glucosidase [EC:3.2.1.20]
K02342	Mar	3.120	0.006	DNA polymerase III subunit epsilon [EC:2.7.7.7]
K00948	Mar	3.118	0.005	Ribose‐phosphate pyrophosphokinase [EC:2.7.6.1]
K00575	Mar	3.114	0.006	Chemotaxis protein methyltransferase CheR [EC:2.1.1.80]
K00140	Mar	3.110	0.019	Methylmalonate‐semialdehyde dehydrogenase [EC:1.2.1.27]
K00134	Mar	3.106	0.021	Glyceraldehyde 3‐phosphate dehydrogenase [EC:1.2.1.12]
K02557	Mar	3.106	0.022	Chemotaxis protein MotB
K02556	Mar	3.093	0.035	Chemotaxis protein MotA
K01999	May	3.668	0.010	Branched‐chain amino acid transport system substrate‐binding protein
K01996	May	3.445	0.008	Branched‐chain amino acid transport system ATP‐binding protein
K01997	May	3.431	0.007	Branched‐chain amino acid transport system permease protein
K01998	May	3.425	0.006	Branched‐chain amino acid transport system permease protein
K01995	May	3.376	0.028	Branched‐chain amino acid transport system ATP‐binding protein
K02433	May	3.316	0.007	Aspartyl‐tRNA(Asn)/glutamyl‐tRNA (Gln) amidotransferase subunit A [EC:6.3.5.6 6.3.5.7]
K02051	May	3.303	0.006	Sulfonate/nitrate/taurine transport system substrate‐binding protein
K01768	May	3.283	0.008	Adenylate cyclase [EC:4.6.1.1]
K02050	May	3.280	0.041	Sulfonate/nitrate/taurine transport system permease protein
K00058	May	3.126	0.025	D‐3‐phosphoglycerate dehydrogenase [EC:1.1.1.95]
K01524	May	3.114	0.025	Guanosine‐5′‐triphosphate, 3′‐diphosphate pyrophosphatase [EC:3.6.1.40]
K01915	May	3.102	0.017	Glutamine synthetase [EC:6.3.1.2]
K01952	May	3.086	0.040	Phosphoribosylformylglycinamidine synthase [EC:6.3.5.3]
K00681	May	3.070	0.011	Gamma‐glutamyltranspeptidase [EC:2.3.2.2]
K01802	May	3.045	0.046	Peptidylprolyl isomerase [EC:5.2.1.8]

Functional profiles of microbial communities associated with *P. lutea* during heat stress were characterized by the enrichment of functions related to amino acid metabolism (Table 2). Several proteins involved in amino acid transport system (permeases and ATP‐binding proteins) were overrepresented in the microbiomes associated with corals experiencing thermal stress. We observed an enrichment in guanosine‐5′‐triphosphate,3′‐diphosphate pyrophosphatase, an enzyme that catalyzes the conversion of pppGpp (guanosine‐5′‐triphosphate,3′‐diphosphate) to ppGpp (guanosine 3′,5′‐bis(diphosphate)), a cytoplasmic signaling molecule that controls the “stringent response,” an adaptive process that allows bacteria to respond to amino acid starvation (Hara & Sy, 1983; Hauryliuk, Atkinson, Murakami, Tenson, & Gerdes, 2015). Under optimal conditions, coral hosts may be sharing nutrients with their associated microorganisms. However, an increase in seawater temperature may trigger the breakdown of this symbiotic relationship and as a consequence, the associated microbes may be experiencing an amino acid starvation. Recently, Zhou et al. (2017) demonstrated that five gene ontology (GO) terms overrepresented in heat stressed *P. damicornis* were related to TNF signaling pathway, apoptosis and cell death (Zhou et al., 2017). Similar results were also reported in *Acropora aspera* (Rosic et al., 2014), *A. hyacinthus* (Seneca & Palumbi, 2015) and *Orbicella faveolata* (Pinzón et al., 2015). Amino acids released from bleached coral tissues undergoing apoptosis and cell death are likely scavenged by their associated microbes. This amino acid scavenging activity appears to be congruent with the observed enrichment in amino acid transport proteins in the functional profiles of microbial communities associated with corals during thermal stress.

A number of studies have highlighted the importance of coral‐associated microorganisms and their roles in fitness and survival of the host animals (Ainsworth et al., 2015; Bourne et al., 2008; Gilbert et al., 2012; Tout, Siboni et al., 2015; Vega Thurber et al., 2009; Ziegler et al., 2017). Nonetheless, prior to this work, very little was known about the effects of thermal stress on the dynamics of *P. lutea‐*associated *Symbiodinium* and bacterial communities. Our study thoroughly examined the shifts in both bacterial and algal populations associated with *Porites* corals during a natural bleaching event. While there were minimal changes observed in *Symbiodinium* community diversity and composition, we provided the evidence of dramatic shifts in structure and diversity of associated bacterial communities when corals were exposed to elevated seawater temperature. The information on how coral‐associated microbiomes shifts their compositions between pre‐bleaching, bleaching, and post‐bleaching stages may help us further our understanding on how these associated bacterial contribute to the resilience of their coral host to thermal bleaching.

## CONFLICT OF INTEREST

The authors declare no competing financial or conflict of interests.
