# Supplementary figures and images for: Dynamics of coral‐associated microbiomes during a thermal bleaching event
Source: Microbiologyopen. 2018 Mar 23;7(5):e00604. doi: 10.1002/mbo3.604 (PMC6182559; doi:10.1002/mbo3.604)

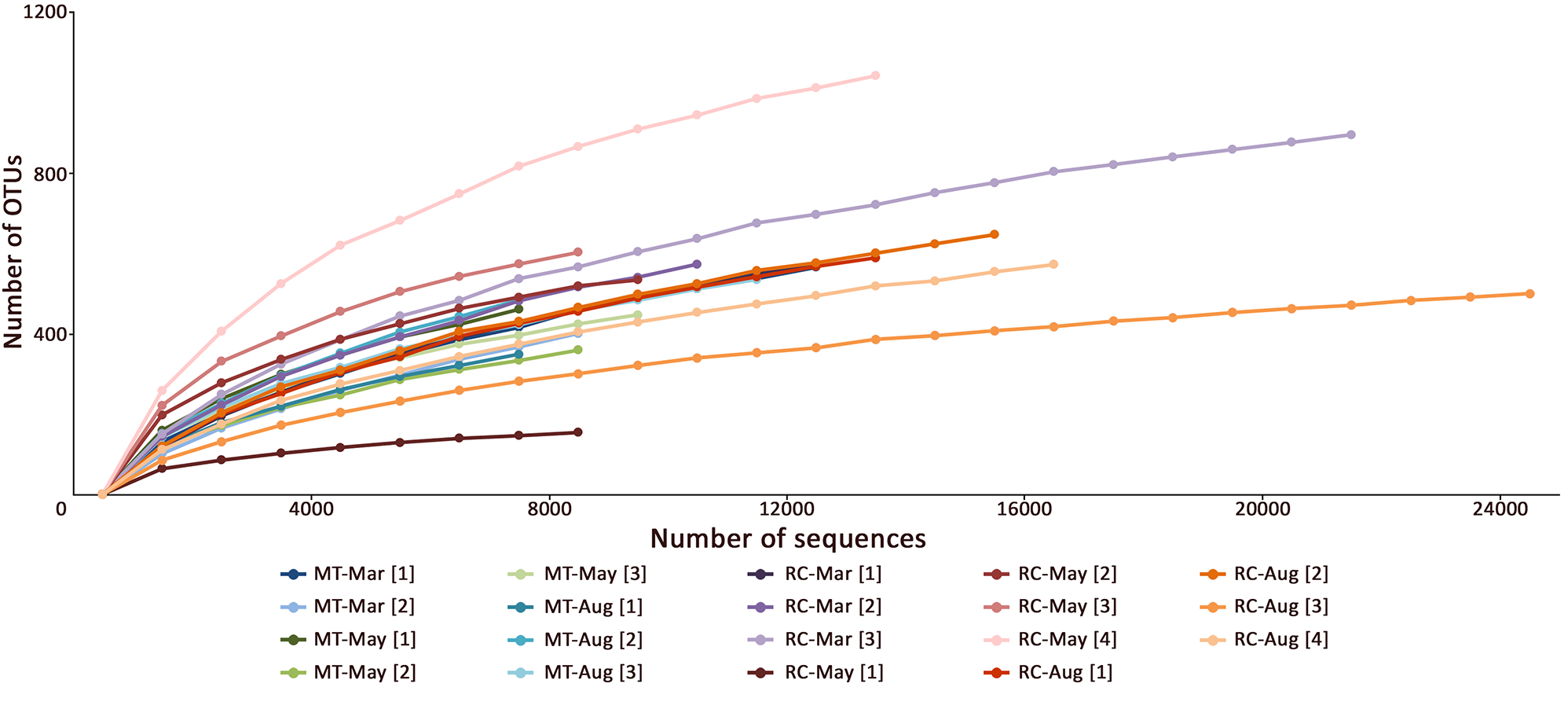

Supplement: Supplementary file 2 [file MBO3-7-e00604-s002.tif]

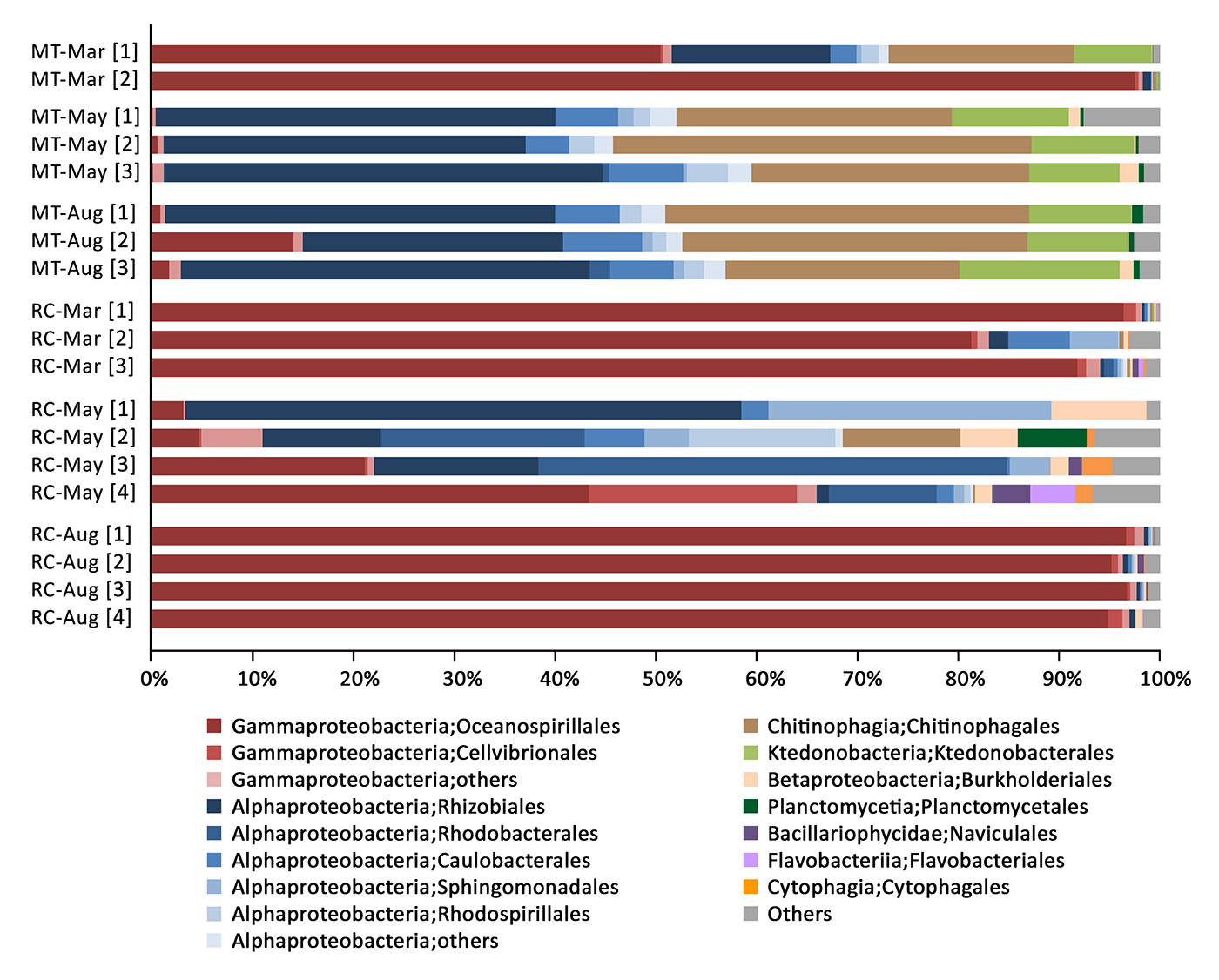

Supplement: Supplementary file 3 [file MBO3-7-e00604-s003.tif]

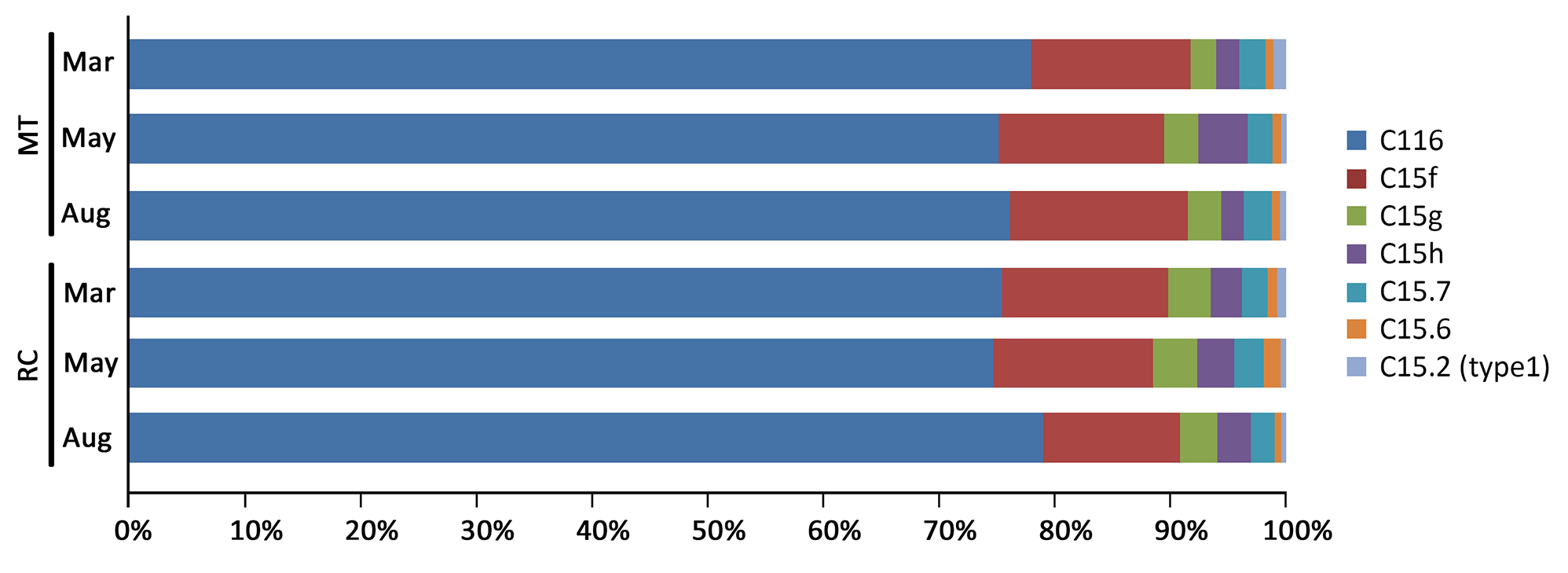

Supplement: Supplementary file 4 [file MBO3-7-e00604-s004.tif]
